# Carving Nature at Its Joints: A Comparison of CEMI Field Theory with Integrated Information Theory and Global Workspace Theory

**DOI:** 10.3390/e25121635

**Published:** 2023-12-08

**Authors:** Johnjoe McFadden

**Affiliations:** Faculty of Health and Medical Sciences, University of Surrey, Guildford GU2 7XH, UK; j.mcfadden@surrey.ac.uk

**Keywords:** consciousness, cemi, IIT, GWS, electromagnetic, theory, integration, information, workspace, AI

## Abstract

The quest to comprehend the nature of consciousness has spurred the development of many theories that seek to explain its underlying mechanisms and account for its neural correlates. In this paper, I compare my own conscious electromagnetic information field (cemi field) theory with integrated information theory (IIT) and global workspace theory (GWT) for their ability to ‘carve nature at its joints’ in the sense of predicting the entities, structures, states and dynamics that are conventionally recognized as being conscious or nonconscious. I go on to argue that, though the cemi field theory shares features of both integrated information theory and global workspace theory, it is more successful at carving nature at its conventionally accepted joints between conscious and nonconscious systems, and is thereby a more successful theory of consciousness.

*“And once I had recognized the taste of the crumb of madeleine soaked in her decoction of lime-flowers which my aunt used to give me (although I did not yet know and must long postpone the discovery of why this memory made me so happy) immediately the old grey house upon the street, where her room was, rose up like the scenery of a theatre to attach itself to the little pavilion, opening on to the garden, which had been built out behind it for my parents (the isolated panel which until that moment had been all that I could see); and with the house the town, from morning to night and in all weathers, the Square where I was sent before luncheon, the streets along which I used to run errands, the country roads we took when it was fine. And just as the Japanese amuse themselves by filling a porcelain bowl with water and steeping in it little crumbs of paper which until then are without character or form, but, the moment they become wet, stretch themselves and bend, take on colour and distinctive shape, become flowers or houses or people, permanent and recognisable, so in that moment all the flowers in our garden and in M. Swann’s park, and the water-lilies on the Vivonne and the good folk of the village and their little dwellings and the parish church and the whole of Combray and of its surroundings, taking their proper shapes and growing solid, sprang into being, town and gardens alike, all from my cup of tea.”*

Marcel Proust, *Memories of Things Past*, 1913

## 1. Introduction

In the above famous lines, Proust’s contemplates the nature of memory but his beautiful prose also illustrates the most profound and characteristic feature of consciousness that, in an instant of perceptual time, the conscious mind can grasp the complexity of the imaginary town of Combray with its walks, flowers, gardens, people and even “the old grey house upon the street, where her room was, [which] rose up like the scenery of a theatre”. Since Descartes, philosophers, scientists and writers have pondered where this ‘theatre’, as it is often described, is within the three pounds or so of grey flesh that inhabits our skull. In recent decades, the observer has been expelled from the Cartesian theatre [1] but there remains the puzzle of locating the theatre itself, the conscious mind, amongst the tangle of 86 billion or so neurons in the brain.

Consciousness is notoriously difficult to define; but then, so is life, yet that has not inhibited biologists from studying the phenomenon. Nevertheless, from the perspective of an article focussed on theories of consciousness, it will be useful to propose a definition. I propose that a conscious system is one in which physically integrated information sufficiently complex to encode thoughts, such as the town of Combray, is generated by a system and acts on that same system to drive motor actions that report thoughts and confer agency on that system.

There are many theories of consciousness (ToCs) many of which have recently been reviewed and compared [2,3,4] but none is universally, or even predominantly, accepted as being an effective description of consciousness. In his pioneering book, “*The Astonishing Hypothesis*” [5], the Nobel Prize laureate, Francis Crick, proposed a revolutionary approach to locating the ‘seat of consciousness’ by identifying measurable neural correlates of consciousness, or NCCs. This approach has been very fruitful in identifying many neural underpinnings of consciousness and anatomical sites in the brain that appear necessary for consciousness but has not yielded any obvious *seat of consciousness* [6]. Brain injury, stimulation and surgical resection studies have similarly identified regions of the brain, such as the thalamus, that appear to be required for conscious experience, and others, such as the cerebellum, whose activities do not appear to be associated with conscious experience but, once again, without identifying an anatomical site or process that is a plausible seat of consciousness [7,8].

The inability of NCC approaches to associate consciousness with a particular neurological process or anatomical site in the brain has led other researchers to tackle the problem from a phenomenological direction to identify properties common to the conscious experience and then uncover what kind of processes or activities are needed to account for these properties. Chief amongst these core consciousness properties is, as Proust’s text illustrates, the ability of the conscious mind to grasp complex perceptions or ideas, such as the imaginary town of Combray, within a singular conscious state. The binding problem is that of understanding how information that is distributed across many millions or billions of neurons throughout the brain is bound into a singular conscious state. Possible solutions to this problem are provided by the two of the most popular ToCs, integrated information theory (IIT) and global workspace theory (GWT). The aim of this review is to examine an alternative solution, the cemi field theory, that, quite simply, places the seat of consciousness in the physical but immaterial electromagnetic field (EMF) generated by brain neural activity.

The idea that consciousness requires some kind of substrate capable of integrating information goes back at least as far as the Gestalt school of psychology that emerged in the early 20th century [9]. Gestalt psychologists insisted that perception and consciousness involve the integration of sensory information, such as the details of the imaginary town of Combray, into complex but integrated wholes which they called “gestalts”. One of the movement’s founders, Wolfgang Köhler, proposed that these gestalt properties of object perception are encoded in electrochemical “brain-field[s]” that are isomorphic with “the field of a percept” [10]. This idea fell out of favour with the emergence of modern neurobiology that did not accept any a role for electric fields in the brain, despite the fact that the brain was known to generate an EM field since Hans Berger’s invention of electroencephalography (EEG) in the 1920s. In the 1990s, philosopher Karl Popper and colleagues [11,12] and, though a little later, the pioneering experimental neurophysiologist, Benjamin Libet [13,14], took up the gestalt field idea to propose that consciousness is provided by some kind of unifying field in the brain. However, neither of these researchers identified consciousness with any known physical field so its nature remained mysterious.

In 2000, both McFadden [15] and Pocket [16] published books that proposed a possible solution. They pointed out that neuron firing and synaptic transmission in the brain generates an endogenous electromagnetic field, as detected by EEG and MEG (magnetoencephalography) studies. Interesting, EEG was also routinely utilized in medical diagnostics to assess states of consciousness in patients. Yet the brain’s EM field was mostly considered to play no role in information processing in the brain.

However, being a field-mechanical entity, brain EM fields are subject to both constructive and destructive interference. Asynchronously firing neurons will generate non-overlapping EM field waves that will tend to cancel each other out by destructive interference. Yet, if neurons are firing synchronously so that the peaks and troughs of their wave oscillations overlap, then the EM field perturbations generated by their firing, and, thereby, the information encoded by the synchronously firing neurons will reinforce each other through constructive interference. So, the brain’s EM field—as detected by EEG or MEG—will be dominated by signals generated by synchronously firing neurons. Numerous studies had, by this time, demonstrated that consciousness in both man and animals is strongly correlated with synchronously firing neurons [5,17,18,19,20,21,22,23,24]; although highly synchronized neuronal firing, as in epileptic seizures, is associated with loss of consciousness, a point to which I will return to later in this article. However, and crucially, whereas information encoded in neurons is discrete and localized, information encoded in brain EM fields is physically integrated and delocalized across the entire brain. In papers published in 2002, McFadden and Pockett (separately) proposed that consciousness correlates with synchronously firing neurons because the substrate of consciousness is not brain matter but the equally physical but immaterial brain’s EM field [25,26,27]. So, according to the EM field theories of consciousness (EMF-ToCs), the town of Combray in Proust’s story would not only be encoded in neurons scattered across the narrator’s brain but also in the EM fields generated by those same neurons firing synchronously so that their encoded information is integrated and broadcast to any potential (receiver) neuron localized anywhere in the brain. EMF-ToCs propose that neuronal information becomes conscious only when it is integrated into brain EM fields.

A key difference between McFadden and Pocket’s theories is that, in Pockett’s theory, the brain’s EM field is proposed to be causally inactive, whereas in cemi field theory, the brain’s EM field is proposed to influence neural firing sufficient to be the cause of motor actions that we experience as willed (rather that automatic) actions, the outputs of the conscious mind [28]. There is considerable evidence, as I recently reviewed [29], that external EM fields, of similar strength and dynamics as brain endogenous fields, do indeed influence neural firing. The threshold EM field perturbation that is needed to influence neural firing is currently unknown, but is likely to depend on a number of factors, such as the resting potential and anatomy of a particular neuron and its environment. However, as I have previously argued, to influence neural firing, the minimum voltage delivered by the brain’s endogenous EM field should at least higher than thermal voltage fluctuations across the membrane, which I previously estimated to be around 13 μV across a 5 nm cell membrane [25]. This value is well within the range of measured extracellular local field potentials (LFPs), which are generally in the range of 1–1000 µV.

Similar EMF theories of consciousness (EMF-ToCs) were proposed around the same time by the neurophysiologist E. Roy John [30,31] and the neurophysiologists Fingelkurts and Fingelkurts [32,33,34]. In the following decades, several other EMF-ToCs have been proposed [35,36,37,38,39,40,41,42,43] and abundant evidence has emerged for EMF-mediated information transfer in the brain, sometimes known as ephaptic transmission [24,44,45,46,47,48,49], consistent with the cemi field’s assertion that the brain’s EM field, the substrate of consciousness, is indeed causally active.

An alternative approach to integrating conscious information in the brain was pioneered by the neurobiologist, Giulio Tononi, who, in 2004, proposed that any system with highly integrated information generates consciousness [50,51]. In contrast to the EMF-ToC’s that propose that conscious information is integrated within a physical field, Integrated Information Theory, or IIT, instead envisages that neuronally encoded brain information becomes conscious only when it is maximally integrated, in the sense of its cause-effect structure being maximally irreducible to cause–effects relationships of the parts of that system. This irreducibility can be evaluated by calculation of the value of a mathematical parameter called Φ (phi), which reflects the irreducibility of a system’s cause-effect structure. The cause–effect network that possesses the highest value of Φ will be conscious. So, the theory would posits that if it were possible to calculate the value of Φ for all the neural networks in Proust’s narrator’s brain, then under the influence of the madeleine cake, that value would be maximal for those that encoded features of the town of Combray. The theory has gathered a lot of support but has also recently been subject to several strong criticisms [52,53].

Global workspace theory (GWT) provides a different phenomenological starting point, which is that of understanding how information encoded in disparate regions of the brain is accessed by the conscious mind in the process that we call thinking, such as when Proust’s narrator thinks about the town of Combray. GWT proposes that consciousness arises from a ‘global workspace’ wherein disparately-encoded brain information can be pooled and integrated where it becomes accessible to working memory, the cognitive system responsible for temporarily holding and manipulating information that is involved in numerous conscious cognitive tasks, such as problem-solving, language comprehension, reasoning, decision-making and writing [54,55]. In GWT, all neuron-encoded information in the brain competes for access to the global workspace, but only the fraction of brain information that succeeds in gaining access to that workspace becomes conscious and is able to deliver actions. The workspace is hypothetical in standard GWT as the theory does not identify the physical nature of the global workspace nor provide insight into the nature of the competition that determines access to the workspace, so the theory does not lend itself to experimental verification. However, its subsequent development as global neuronal workspace theory (GNWT) by Dehaene and Changeux [54,56,57,58,59,60] introduced an experimentally detectable ‘ignition’ component associated with a temporary increase in synchronized firing leading to a coherent interconnected network of neuronal activity proposed to act as a kind of dynamic global workspace that distributes its neuronally encoded information to the entire brain. So, according to GNWT, if we could examine the brain of Proust’s narrator whilst he was eating his madeleine cake then you would find a network of synchronously firing neurons encoding features of the town of Combray. A recent study of magnetoencephalography (MEG) patterns in human subjects examined information flow between brain regions involved in performance of seven different tasks. The study identified brain regions including the precuneus, posterior and isthmus cingulate, nucleus accumbens, putamen, hippocampus and amygdala, which were active across all seven tasks and thereby consistent with being a global neuronal workspace that orchestrates information from perceptual, long-term memory, evaluative and attentional systems [61] to deliver actions such as completing the test tasks or writing a novel.

In this article, I assess the CEMI field theory together with IIT and GWT/GNWT against the Platonic ideal of science as carving nature at its joints.

## 2. Carving Nature at the Joints

Plato is said to have described the job of philosophy, and by modern extension, science, as that of *carving nature at the joints*, essentially finding those features that distinguish objects according to their kind or form [62]. For example, a theory that identified dogs as animals that bark and wag their tails and cats as animals that purr would be a good theory as it, at least, carves the animal world between dogs and cats. An alternative theory that identified dogs as animals with claws would be a bad theory since it would also identify cats or bears as dogs. From the perspective of this article, a ToC should be able to distinguish between objects or systems that are conscious and those are that are nonconscious. Of course, there is a great deal of controversy on where the division between conscious and nonconscious living organisms lies but the consensus amongst scientists, philosophers and the rest of humanity is that humans are conscious along with many mammals, such as primates, dogs and cats, but plants, microbes and inanimate objects such as toasters, computers, rocks, photodiodes or electrical grid systems are not conscious.

The criteria used to distinguish between conscious and non-conscious entities are, of course, anthropomorphized and thereby problematic. We tend to infer that objects that behave somewhat like ourselves, such as dogs and cats, are conscious, whereas objects that behave very differently, such as toasters, plants or rocks, are not conscious. Yet the inference is not without foundation. Human consciousness is perceived to be the driver of what we call “free will” [28], the impression that some of our actions are driven by our conscious choices or *agency*, rather than automatic responses. It seems reasonable to infer that dogs, cats, primates and other higher animals that are generally considered to possess at least a degree of agency may also be conscious. Rocks, toasters, plants and computers that lack signs of agency are considered to also lack consciousness. I argue here that any alternative carving of nature into conscious and nonconscious systems predicted by a ToC should, at the very least, be supported by independent evidence for its alternative carving, for example, signs of consciousness in systems that are generally considered to be nonconscious but are predicted by the ToC to be conscious.

A related criterion is the need to distinguish between nonconscious and conscious information processing within a single human brain. It is well established that most of what the brain does is non-conscious, irrespective of its level of complexity. For example, the processing of sensory information to direct the delicate movements that are involved in keeping us upright when walking, running, playing tennis, etc., are highly complex, yet are performed automatically without conscious awareness most of the time. Similarly, we do not need to consciously think about the delicate and complex movements of our larynx, lips and tongue needed to form words when speaking. Even semantic processing, such as formulating spoken sentences in their correct grammatical constructions, is performed without awareness: we are not aware of the fine-grained and complex grammatical rules that our brain automatically applies to order and conjugate words into well-formed sentences. Evidence from brain imaging studies suggest that these non-conscious cognitive feats involve large distributed regions of the brain, and thereby highly complex neural processing, but without awareness [63,64]. Yet, we can be painfully aware of very simple, unintegrated stimuli, such as when someone stands on our toe. A successful ToC should account for the differences between conscious and nonconscious information processing in the brain.

Moreover, highly complex and active several specialist structures in the brain, such as the cerebellum, appear to operate without consciousness. For us humans at least, we are aware only of the comparatively sparse contents of our conscious mind, more of a trickle rather than a stream of consciousness. Any ToC should thereby be able to carve nature between the brain’s torrents of nonconscious neural processing and their adjacent conscious trickle. Additionally, a successful ToC should also be able to distinguish between conscious and nonconscious states in the entire brain, such as between waking and deep sleep or anaesthesia.

## 3. Global Workspace Theory

The cognitive neuroscientist, Bernard Baars, first proposed GWT in his book ‘A Cognitive Theory of Consciousness’ [65] published in 1988 to account for how the brain selectively attends to certain information while ignoring other stimuli. Its central idea is a hypothetical global workspace where information from distributed neurons across the brain is pooled, integrated and made available for the delivery of conscious outputs, such as speech or writing. Since the global workspace—broadly equivalent to working memory—has a limited capacity, there is competition amongst neural networks to gain access to its ‘theatre’ to highlight, by a kind of attentional spotlight, and make available, a neural audience of motor outputs such as those involved in speech or writing.

Since GWT defines the global workspace functionally, rather biologically, it is not clear whether the presumed restriction of consciousness in GWT to biological brains is valid. Many animate or inanimate system could also be defined as accessing a functional global workspace. For example, the bloodstream pools and transmits a wide variety of information sources, such as hormones, cytokines, chemokines and nutrients to the cells of the body and could thereby be considered as a circulatory global workspace. Similarly, the air around us pools and transmits lots of information encoded in acoustic vibrations generated by spoken language that it makes available to anyone in within earshot. Computer memory systems, such as Random Access Memories, also act as global workspaces, just as the internet acts as a global workspace accessible to anyone with a computer or smartphone. ChatGPT could be even be considered as the mouthpiece of the internet’s global workspace. But none of these electronic systems are generally considered to be conscious.

If possession of a global workspace is sufficient for consciousness, then GWT, is rampantly panpsychist. GWT theorists generally sidestep this problem by framing GWT within cognitive neuroscience and specifically within the brain, or even regions of the brain such as the prefrontal cortex and other regions involved in higher-order cognitive functions. But then what is special about brain global workspaces that, rather than non-brain global workspaces, makes them conscious? This is not addressed within GWT, so, GWT does not carve nature at the common-sense joints between conscious and non-conscious systems, except by unspoken criteria that distinguish between conscious and nonconscious systems global workspaces. This moves the differentiation between conscious and non-conscious systems away from GWT towards some—as yet unformulated—theories of why only brain global workspaces are conscious. GWT is then not really a theory of the nature of consciousness but one of its function.

However, GWT does succeed in dealing with nonconscious states, such as anaesthesia. It proposes that neural information may be restricted from entering or leaving the global workspace during non-conscious states. This allows information or cognitive processes to be active in the brain, but not part of the conscious experience. GWT does not predict the correlation between consciousness and synchronously firing neurons. However, Global Neuronal Workspace Theory (GNWT) does propose that global ignition events, which are proposed to be responsible for distributing global workspace information around the brain, are generated by synchronously firing neurons. So, GNWT does correctly predict that consciousness will be correlated with synchronously firing neurons. However, GWNT does not, in itself, provide a mechanism by which the brain can distinguish between synchronously firing neurons from those that are not synchronously firing. Even in ignition events, most neurons in the brain are not firing synchronously, so how does the brain *know* which neurons are firing synchronously and thereby constitute the global workspace to be used in conscious thinking? This is not specified in GNWT.

Regarding the nonconsciousness of the cerebellum, in some papers, Baars and colleagues identify the likely site for the global workspace as the “cortico-thalamic core [which] is a great mosaic of multi-layered two-dimensional neuronal arrays. Each array of cell bodies and neurites projects to others in topographically systematic ways” [66]. Baars goes on to argue that “this connectivity is different from other structures that do not directly enable conscious contents, like the cerebellum. The cerebellum is organized in modular clusters that can run independently of each other, in true parallel fashion. But in the C-T [cortico-thalamic] core any layered array of cortical or thalamic tissue can interact with any other, more like the world-wide web than a server farm” [66]. But the authors do not explain why the “world-wide web-like” global workspaces support consciousness, whereas ‘great mosaic[s] of multi-layered two-dimensional neuronal arrays’, as in the cortico-thalamus, do not support consciousness. Also, why the world-wide web itself is not conscious is not explained within GWT. Instead, the division between conscious and non-conscious systems in the brain is not provided by GWT itself but by additions to the theory. The theory itself does not carve nature at its generally accepted joints between conscious and nonconscious systems.

## 4. Integrated Information Theory

The starting point for Integrated Information Theory [50,67] (IIT) is a set of five axioms. The first three, that consciousness exists, that consciousness is compositional or structured and that consciousness is information-rich, are uncontroversial; although a key point regarding the third axiom is that conscious information is said to be intrinsic in the sense that it is independent of the observer. The fourth and fifth axioms are what defines IIT. The fourth axiom is that consciousness is integrated. Specifically, in his 2008 IIT ‘Provisional Manifesto’ Tononi insisted that ‘*to generate consciousness, a physical system must be … unified; that is, it should be doing so as a single system, one that is not decomposable into a collection of causally independent parts’* [68].

In IIT, information in information processing systems, such as the brain, may be integrated through its “the cause-effect structure” which, in IIT, describes the complex network of interactions and dependencies among the elements or components of the system. IIT evaluates how elements within a system causally affect one another in causal relationships that can be complex and involve feedback loops and interactions in multiple directions. Importantly, IIT is a quantitative theory that uses a mathematical measure, known as Φ (phi), to represent the extent to which the system’s cause–effect structure is irreducible, meaning that it cannot be explained by looking at individual elements in isolation. Φ is calculated for all possible subsets of elements within the system, from a single element to the entire system. For example, in the context of the human brain, subsets could include individual neurons, groups of neurons, or larger brain regions, such as the cerebral cortex or the cerebellum. Since, in a set of *n* elements there are 2*^n^* possible subsets, for a brain of around 10^11^ neurons, there are potentially 2 to the power of 10^11^ subsets that need to be evaluated, which is clearly impossible for the brain or indeed any realistic information processing system. Consequently, IIT is more concerned with the likely behaviour of Φ within an information processing system, rather than any attempt to calculate its exact value for those systems.

IIT theory proposes that subsystems with high Φ values, and thereby more integrated and interconnected cause–effect structures, have the potential to be conscious. Clearly, in any complex information processing systems, such as the brain, there would be a huge number of overlapping system subsets with high values of Φ and thereby an astronomical number of potentially conscious subsystems. To avoid a combinatorial explosion of competing and overlapping consciousnesses, IIT includes a fifth “exclusion” axiom, which states that “A mechanism can contribute to consciousness at most one cause–effect repertoire, the one having the maximum value of integration/irreducibility Φ [67]. So, in IIT, all elements of a system and all possible partitions of these elements are considered, but the one that maximizes Φ is considered to be conscious. All sub-maximally integrated information within the same system is proposed to be nonconscious: “Of all overlapping sets of elements, only one set can be conscious—the one whose mechanisms specify a conceptual structure that is maximally irreducible (MICS) to independent components” [69]. So, IIT carves neural processing between a singular consciousness and multiple nonconscious streams in the brain.

In the IIT context, information is considered “intrinsic”, meaning that it can be defined independently of a particular observer or reference frame. This does, however, generate a problem, since, as Barrett and Mediano have argued, “however, one might reformulate the theory, any attempt to create a formula for consciousness as intrinsic information needs to define, spatially, where one system ends and another begins.” [70]. Any attempt to calculate a Φ-like parameter is thereby observer-dependant. Indeed, as Barrett and Mediano [70] demonstrated, Φ is ill-defined and thereby mathematically impossible to calculate for any system. It has only been computed for toy model systems in an imaginary universe composed of indivisible binary components that evolve in discrete time with a well-defined transition probability matrix. Giulio Tononi counters that, just as the planets do not need to calculate their trajectories through space, nature does not need to calculate Φ for systems that possess its highest value to be conscious.

Yet, the systems are not equivalent. As Tononi argues, from an intrinsic perspective, a planet does not need to calculate its orbit; instead, the calculation is *implemented* in the interaction of the planet’s mass with the gravitational system of the entire solar system. But then the biggest planet in the solar system does not possess a novel property distinct from smaller planets, such as consciousness. If it did, then that property would not be explained by size, mass or gravity alone, as all the planets possess these to some degree. Similarly, consciousness cannot be accounted solely by Phi if only systems with its highest value are conscious. Some additional factors are necessary to assign a conscious value of 1 to the system with the highest Φ, and zero to all other systems.

Perhaps a better analogy is with phase transitions when, for example, ice becomes water, and water becomes steam as temperature increases. But, in this case, temperature (analogous to Φ) does not predict the phase transition (analogous to emergence of consciousness) from first principles. Instead, molecular interactions, such as Van der Waals forces, hydrogen bonding and ionic interactions, also have to be taken into account. Similarly, for consciousness within a system to be singular, Φ alone cannot account for consciousness. Other factors must be involved that separate the Φ winner from the runners-up.

Although Φ cannot be calculated, it is, as I have previously highlighted [28], essentially a variant of *mutual information* which, in probability and information theory, is a measure of the mutual causal dependence between different variables within a system [71],and, like Φ, has been used to assess levels of consciousness in patients [72]. Unlike Φ, mutual information can be and has been calculated for a wide variety of complex systems, ranging from social networks [73,74] to communities [75,76], ecological networks [77], and financial [78] and institutional networks [79]. Systems biology approaches have calculated mutual information for physiological systems [80], transcriptional regulatory networks, immune networks [81], metabolic networks [82] as well as brains [72]. These systems are also highly complex. For example, the immune system is composed of around 10^12^ interacting immune cells, slightly more than the brain’s 10^11^ interacting neurons. Moreover, whereas neurons primarily communicate via a handful of neurotransmitters, immune cells communicate via hundreds of chemokines and cytokines. All of these systems are characterized by high levels of causally integrated complex mutual information that are thereby highly likely to be “not decomposable into a collection of causally independent parts”, and thereby highly likely to include subsystems with high values of Φ, if Φ could be calculated. Similarly, high values for mutual information have also been described for artificial intelligence systems, such as in robotics [83] deep neural networks [84] as well as random Boolean networks [85]. Each of these systems will potentially have some partition with a maximally high value of Φ which would, according to IIT, be conscious. Yet the consensus amongst immunologists, cell biologists, molecular biologists, systems biologists and computer scientists is that none of these systems are conscious. Of course, they could be wrong, but I believe it is up to proponents IIT to provide independent evidence for the predictions of their theory.

It should similarly be possible to find a maximally high value of Φ for any information processing system, including all living organisms from bacteria to plants and animals, as well as inanimate devices, including, not only computers, but even photodiodes or large-scale electrical power grids [53,86]. IIT is thereby, like GWT, hugely panpsychist. Tononi accepts the rampant panpsychism implied by IIT and goes on to argue that any inanimate system that can process information in a highly interconnected and irreducible manner will, in theory, exhibit some degree of consciousness, irrespective of its cognitive capabilities: what he refers to as being ‘noncognitively conscious’ [86]. Tononi does, however, insist that simple systems, such as photodiodes, are likely to possess only a very limited conscious experience, very different from the rich phenomenology of an adult human brain. But then the distinction between conscious and non-conscious entities, according to common sense, is not accounted for within IIT, but, instead, must be attributed to some, as of yet, unspoken theory that differentiates between cognitive and non-cognitive conscious states. IIT alone is clearly unable to carve nature at the joints between systems that are generally accepted to be conscious and non-conscious entities.

IIT performs better in distinguishing between conscious and non-conscious neural activity within the brain by proposing that they differ in terms of their level of complexity and integration, as measured by Φ. For example, a proxy for Φ has been estimated and used to examine EEG patterns (as a surrogate for detailed knowledge of neural firing patterns), which was quite successful in distinguishing conscious and non-conscious patients with disorders of consciousness (DoCs) [51,87] and anaesthesia [88]. However, it should also be noted that EEG is the most widely-used tool for clinical assessment levels of consciousness in patients with DoCs or anaesthesia [89], and most applications do not apply IIT but use simpler measures such as information complexity or (negative) entropy [90,91].

The proponents of IIT have claimed that IIT successfully predicts that the cerebellum is nonconscious (as is generally believed) on the grounds that its values of Φ are likely to be relatively low compared to the rest of the brain [69]. However, this conclusion was based entirely on analysis of toy networks used to represent the cerebellum consisting of only 12 elements grouped into three “modules” [50,92]. As pointed out in a 2022 paper [86], this toy network grossly underestimates the enormous complexity of the cerebellum with its 69 billion neurons (more than half of the neurons in the entire brain) together with several internal structures, such as its four pairs of cerebellar nuclei that receive and transmit signals from sites both within and outside of the cerebellum. No actual calculation of Φ has ever been attempted for the cerebellum, nor would it be possible, even in principle, so there is no evidence that the cerebellum is associated with relatively low values of Φ, or any Φ proxy.

However, in a 2023 paper [93], Tononi and colleagues appear to allow for the possibility that the cerebellum is sufficiently complex to be associated with a maximal value of Φ that would confer a degree of consciousness writing that “what can also exist [in the brain] are other intrinsic entities, likely small, whose substrates do not overlap with the main complex [the conscious part of the brain with its highest value of Φ]. These many small Φ-structures are mere ontological “dust” … unfolded from, say, groups of neurons arranged in partially segregated loops in prefrontal areas or in the cerebellum”. I do not understand what is meant by ‘ontological dust’ in this statement, but for any part of the cerebellum to be independent (from reportedly conscious regions of the brain) according to IIT’s exclusion principle, its cause–effect structure must “not overlap with the main complex”. It is difficult to see how this could be possible as the cerebral cortex has multiple connections with nearly all regions of the brain, including those intimately associated with consciousness, i.e., the motor cortex, prefrontal cortex, and parietal cortex. IIT thereby currently fails to account for why the cerebellum is nonconscious.

IIT does have another problem in accounting for nonconscious states of the entire brain, such as in anaesthesia, deep sleep or during epileptic seizures. The problem arises because IIT is built on the premise that those neural networks with the highest values of Φ will be conscious, irrespective of their actual value of Φ. Living brains are never completely idle so it will always be possible to calculate values of Φ for the trillions of partitions of active and inactive neurons. So, brains will always have neural networks with a range of values of Φ including very high values, remembering that, in IIT, inactive neurons also contribute to the conscious state. Just as newspapers never have blank front pages, in IIT, brains will always be conscious. Once again, this does not correspond to our experience or commonsense notion of consciousness.

Another problem concerns the ‘exclusion’ axiom of IIT, which states that if a system S1 overlaps with another system S2 that has higher Φ than S1, then there is no consciousness associated with the part of S1 that does not overlap with S2. However, if the connection between S1 and S2 were to be severed then S1 would become an independent conscious entity. As has been previously noted [86], if S1 and S2 are considered to be the left and right hemispheres of the brain, then the effect of severance between S1 and S2 has been tested in (split-brain) patients who have undergone surgical severance of the principal connection between the two cerebral hemispheres: the corpus callosum. Tonini and Koch have claimed [51] that according to IIT, “there would be an all-or-none change in consciousness: experience would go from being a single one to suddenly splitting into two separate experiencing minds (one linguistically dominant), as we know to be the case with split-brain patients. This would be the point at which Φmax for the whole brain would fall below the value of Φmax for the left and for the right hemisphere taken by themselves”. Early studies [94,95,96] concluded that the severance of the corpus callosum left patient with two consciousnesses, one for each hemisphere, and consistent with IIT’s exclusion axiom and Tononi and Koch’s prediction; however, more recent re-examination of these data, together with new studies [97,98,99], has demonstrated that although visual fields are indeed split in these patients, their consciousness remains intact and unified. Patients respond with either hand to stimuli presented to the right or to the left of fixation in their visual field. This appears to be inconsistent with the predictions of IIT, but is predicted by the cemi field theory as to whether or not a patient’s corpus callosum remains intact, their brain’s EM field remains singular and unified. It should, however, be noted that indirect connections exist between the two hemispheres even after severance of the corpus callosum, therefore, exactly what IIT would predict in this complex is not clear.

## 5. CEMI Field Theory

The proposal that consciousness is the experience of the brain’s EM field has features of both IIT and GWT. Firstly, physical fields automatically (without need for any calculation) physically integrate information. For example, our weight represents an integration of our mass with that of the entire planet performed instantly by the Earth’s gravitational field. EM fields similarly integrate information, for example, the direction of a compass needle represents an integration of the magnetic moment of the entire planet with that of the needle. We are also familiar with the distributed nature of EM field-encoded information whenever, for example, we download a movie from any position within the range of a Wi-Fi router. The CEMI field theory simply proposes that consciousness is the experience of the integrated EM-field-encoded information generated by 80 billion or neurons in the brain.

Note that this physical integration of information in space, as in a gravitational or EM field, is very different from the causal integration described by IIT. For example, the last logic gate in a computer that predicts the weather and outputs “rain” can be considered to causally integrate (in the IIT sense) all of the information that went into that prediction. But because the interconnected trains of logic gates that led to that predicted are separated in time, there is no physical system that corresponds to all of the causally integrated information that went into the calculation. In fact, the final logic gate integrates just a few bits of information from its input nodes. It is an integration in time rather than space [29], and does not correspond to field-based integration where all the components of the information are physically integrated at each point in the space of the field.

Proposing that EM fields are sometimes conscious may seem strange but is it any stranger than proposing that matter is sometimes conscious? As Einstein’s famous equation highlights, matter and energy are equivalent; both are entirely physical. However, whereas information encoded in matter (except in exotic states, such as Bose–Einstein condensates) is discrete and localized, information encoded in EM fields is always integrated and delocalized, exactly what we would expect for the substrate of consciousness.

EM fields are everywhere so CEMI field theory has the same potential for panpsychism as IIT and GWT. However, just as all matter has the potential to be alive, but only a very small subset of that matter possesses the property of life, the CEMI field theory insists that although all EM fields have the potential for consciousness, only a small subset of EM fields are in fact conscious. In CEMI field theory, consciousness is proposed to have evolved when, during the process of evolution, neurons became more and more tightly packed within space-limited hard skulls such that EM field interference began to influence neural firing, so was captured by natural selection to deliver novel capabilities, such as *EM field computing* [25,100]. This is a form of analogue computing in which the computation is performed by the interaction of EM fields, rather than the binary digits that are used to compute both in conventional computers and, as far as we know, in the neuronal mind. Just as von Neuman architecture computation could be implemented in almost any physical system, but is found only in artificially designed devices, so field-based computation could be implemented anywhere but, so far, has only (according to cemi field theory) been found in systems designed by natural selection, such as the human brain.

Sometimes called ‘quantum-like’ computing [100,101], EM field computing confers, according on the CEMI field theory, the capability of computing with gestalt objects, such as the idea of the town of Combray, transmitted into the brain’s EM field—its global workspace—by a synchronously firing neurons. Note that this EM field-based computational capability is entirely lacking in conventional computers that are designed to avoid EM field interference between electrical components. So, the CEMI field theory predicts that conventional computers or electrical devices, such as power lines, are not conscious. Indeed, the theory makes the strong prediction that conventional computers built to exclude EM field interactions will never be conscious. Moreover, since EM field computational devices are unknown in nature outside of brains, the CEMI field theory correctly carves nature at the common-sense joint being a property of the living but not inanimate world.

As outlined above, the CEMI field theory was originally proposed to account for experimental findings that consciousness is correlated with synchronously firing neurons [25], so it correctly associates consciousness with such. The CEMI field theory also accounts for why neural activity in the cerebellum appears to be non-conscious, despite the fact that neuronal oscillations are clearly present [102,103,104]; and typical values for local field potentials (LFPs) can be measured in the cerebellum [105]. The key finding is the invisibility of the cerebellum in EEG or MEG measurements [106]. This indicates that, despite cerebellum LFPs, cerebellum-encoded information is not being broadcast into the brain’s global EM field (as detected by EEG and MEG), which in the cemi field theory is proposed to be the seat of consciousness. The reason for the invisibility of the cerebellum on EEG and MEG is not clear, but may be due to the cerebellum’s intricate folding, compared to cerebral cortex, which ensures that currents arising in neighbouring patches of cerebellum activation tend to be running in opposite directions resulting in cancellation of their EM fields through destructive interference. Another possibility is that unlike the cerebral cortex, the cerebellum may not generate phase-locked oscillations and thereby synchronously firing neurons which project their information into the brain’s EM field-encoded global workspace.

The theory also accounts for the lack of consciousness in absence epileptic seizures in which patients lose consciousness. These are associated with strong regular and usually bilaterally synchronous and symmetric EEG signals particularly in the 2–4 Hz range [107]. Naively, one might expect that that the CEMI filed theory might predict that strong EEG signals would be associated a heightened, rather than reduced state of consciousness. However, in contrast to the information-rich EM-encoded information detectable in a normal EEG, which correlates with sensory information, perception, memory and the contents of consciousness, the highly rhythmic EMF fluctuations, characteristic of EEG seizures, are devoid of information, so they cannot encode thoughts. Information content in signals are often assessed as Shannon information, the number of accessible microstates or entropy of the system. Loss of entropy, associated with highly regular EEG signals, is widely used as a measure to detect the onset of epileptic seizures [108,109]. In EMF-ToCs, the loss of information in EEG represents a kind of consciousness brain-wipe that is entirely consistent with the loss of consciousness in absence seizures.

Also consistent with the CEMI field theory is the fact that EEG and MEG, both measures of brain EM fields, are routinely used to detect signs of consciousness in anaesthesia [110,111,112,113,114] and in disorders of consciousness, such as locked-in syndrome [89,115]. Novel prosthetic devices, including brain–computer interfaces [116,117] that detect EEG signals, have recently been developed to restore communication and control to people paralyzed by chronic neuromuscular disorders and allow locked-in patients to communicate via their (conscious) EEG signals. These advances demonstrate that the information needed to consciously direct the motion of limbs is encoded in brain EM fields. CEMI field theory adds to this necessary conclusion the proposal that brain EM fields also direct the conscious motion of the body in healthy people. Of course, other ToCs sometimes accommodate these developments, but the CEMI field theory is the only ToC that predicts them. Once again, the CEMI field theory correctly carves nature at the commonly recognized joints between conscious and nonconscious neural activity.

According to the CEMI field theory, neurons located anywhere in the brain have access to information encoded in the brain’s EM field that has been put there by synchronously firing neurons. The brain’s CEMI field thereby acts as the brain’s global workspace and is consistent with GWT. Since brain EM field information is mostly a product of synchronously firing neurons, the theory is also consistent with GNWT, identifying neuronal ignition events, or neuronal avalanches as they are sometimes called, as the gateway into the brain’s EM field global workspace.

The CEMI field theory as the physical instantiation of global workspace and thereby the substrate for working memory is also consistent with recent remarkable findings by Pinotsis and Miller [118,119]. In a paper entitled ‘*Beyond dimension reduction: Stable electric fields emerge from and allow representational drift’*, the team examine what is known as *representational drift*. Although standard neurological theories of memory generally propose that memories are encoded in *hardwired* neural ensembles, recent studies have demonstrated instead that the exact neurons maintaining a given memory in working memory actually varies from trial to trial: representational drift [120]. It is clearly difficult to account for representational drift in any neuronal-based theory of working memory but Pinotsis and Miller’s studies reveal that, although the neurons encoding a memory change from trial to trial, stability of working memory emerges at the level of the brain’s electric fields, as detected by EEG [118]. In their 2023 paper [119], the author’s go on to propose that ‘electric fields sculpt neural activity and “tune” the brain’s infrastructure’. The higher level of correlation between the contents of working memory and the brain’s EM fields in these studies, rather than the state of the brain’s matter-based neurons, represents a considerable challenge to all neural-ToCs, but is entirely consistent with the CEMI field theory.

## 6. Conclusions

IIT, the cemi field theory and GWTs all agree that consciousness provides a general workspace where information, such as the details of the imaginary town of Combray, is pooled to be made accessible to those neurons that deliver actions, such as writing. The theories differ in the location of the general workspace and its physical nature. Both the cemi field theory and IIT propose that general workspace is composed of integrated information, but the theories differ in how that is integration is accomplished. IIT proposes that consciousness is associated with those neural networks in the brain that have the highest vales of Φ, a measure of the interconnectedness and irreducibility of each network’s information content. But a question that often arises in criticisms of IIT is whether the nature of the mechanisms in the brain are capable of calculating Φ to allocate different levels of consciousness to different neural networks? When presented with this question, Giulio Tononi has argued that just as the planets themselves do not need to calculate their trajectories through space, nature does not need to calculate Φ. Instead, the neural network associated with the highest values of Φ simply becomes conscious.

This analogy does provide an interesting insight into the physical nature of consciousness. As Tononi insists, the planets do not calculate their own trajectories. However, that calculation is nonetheless undertaken, not by the planets themselves, but by the gravitational field generated by their masses and their motions. It is this real physical field, not an ephemeral ‘nature’, that integrates the information inherent in the masses of entire solar system to determine the planet’s trajectories via its influence (in General Relativity) on the curvature of spacetime in the vicinity of the solar system. If Φ or a similar measure of information integration is indeed analogous to planetary trajectories, then, like mass, it needs to influence a physical field that can integrate conscious information. But which one?

The fundamental particles of matter, including neurons, are, of course, all excitations of fields: the weak and strong nuclear fields together with the EM field and the gravitational field. So, whatever ToC is adopted, irrespective of whether it places awareness in the matter or EM fields of the brain, awareness and consciousness must, ultimately, be a property of one or more of those fields. But which one is capable of integrating neural information to generate the conscious experience of imagining the town of Combray in the way that the gravitational field integrates the mass and motion of planetary bodies to determine their trajectories?

The weak and strong nuclear forces have only very short-ranges and are thereby only capable of integrating information within single atomic nuclei. They cannot integrate information across atoms. This, of course, is why particles of matter have discrete locations in space. Both electromagnetism and gravity do have potentially infinite ranges and so are capable of integrating information across the entire brain. However, as far as we know, there is no net transfer of mass in the brain during neural computations, so the brain’s gravitational field does not reflect neural computations. Moreover, as far as we know, the brain’s gravity does not influence neural firing. So, it cannot provide the substrate for the town of Combray in the brain’s global workspace.

The only physical field that is sensitive to neural firing and has the range to integrate information across the entire brain to provide a substrate for the imaginary town of Combray in the brain’s global workspace, which is also capable of influencing neural firing to provide outputs, such as writing, is the brain’s EM field.

In this article, I have shown that the cemi field theory outperforms both GWTs and IIT in carving nature at the generally accepted joints between conscious and nonconscious systems. Of course, those joints may be mistakenly located, but, as I have argued, any theory that proposes an alternative carving of nature’s joints should provide evidence that is independent of the predictions of their theory. As far as I know, no such evidence is available.

However, as noted above, the cemi field theory does share elements with both GWTs and IIT. Moreover, recent ‘weak’ versions of IIT emphasize the importance of integrated information but with reduced ontological commitment to Φ as the potential substrate of consciousness [121]. Other comparisons between these theories could be undertaken that might discover more common ground. For example, IIT has recently had success in accounting for aspects of the phenomenology of consciousness [122]. Jesse Winters has recently similarly accounted for phenomenological aspects of consciousness, such as temporal continuity, within his own EMF ToC known as Temporally Integrated Causality Landscape (TICL) theory [123]. Progress could perhaps be made by incorporating aspects of all three theories to construct a *grand unified* theory of consciousness.
